# Prospective association of eHealth literacy and health literacy with physical activity among Chinese college students: a multiple mediation analysis

**DOI:** 10.3389/fpubh.2024.1275691

**Published:** 2024-02-08

**Authors:** Hua Xuan Liu, Bik Chu Chow, Holger Hassel, YaJun Wendy Huang, Wei Liang, Run Bin Wang

**Affiliations:** ^1^School of Physical Education and Sport Science, Fujian Normal University, Fuzhou, China; ^2^Provincial University Key Laboratory of Sport and Health Science, Fujian Normal University, Fuzhou, China; ^3^Department of Sport, Physical Education and Health, Hong Kong Baptist University, Kowloon Tong, Hong Kong SAR, China; ^4^Institute for Applied Health Sciences, Coburg University of Applied Sciences and Arts, Coburg, Germany; ^5^School of Physical Education, Shenzhen University, Shenzhen, Guangdong, China

**Keywords:** eHealth literacy, health literacy, physical activity, path analysis, prospective design

## Abstract

**Background:**

The COVID-19 pandemic has decreased physical activity (PA) while increasing demand for electronic health resources. eHealth literacy (EHL) is expected to aid eHealth use and health promotion. EHL was raised on the grounds of health literacy (HL). This study explored the associations among EHL, HL, and PA in Chinese college students and identified mediating mechanisms in the EHL/HL-PA relationship.

**Methods:**

An integrated social-cognitive model was proposed. A total of 947 Chinese college students (52.8% women, age = 19.87 ± 1.68 years) completed the three-wave data collection. Path analysis was performed.

**Results:**

An adequate good-to-fit model was indicated. Perceived EHL (PEHL) was significantly associated with perceived HL (PHL) and HL performance (HLP); PHL was negatively related to HLP; PEHL was significantly associated with self-efficacy (SE) and social support (SS); PHL had a significant effect on SS but not SE; HLP significantly affected SS but not SE; SS and SE positively predicted intention (INT), which then predicted PA. SS mediated PEHL/PHL-INT links; SE mediated the PEHL-INT link; SS and INT jointly mediated PEHL/PHL/HLP-PA; SE and INT jointly mediated PEHL-PA.

**Conclusion:**

Relationships among EHL, HL, and PA were explored with multiple mediating mechanisms identified. Differential SE and SS roles in EHL/HL-PA links suggest new mechanisms to inform EHL/HL intervention development.

## Introduction

As an important component of lifestyle behaviors, regular physical activity (PA) is a population health issue (1) with numerous well-documented benefits (2–5). However, physical inactivity has become widespread among Chinese college students in recent years (6–9). Additionally, the coronavirus disease 2019 (COVID-19) pandemic (10) has led to reduced PA for college students, aggravating the problem of physical inactivity (11). An accumulating body of evidence indicates that physical inactivity significantly increases the risk for obesity, chronic diseases, and adverse health outcomes (12–14). It is essential to promote PA engagement throughout the day. Against this background, it is worth exploring the predictors of PA in this target group.

The COVID-19 pandemic has led to decreased PA but also created opportunities to obtain health information from electronic resources due to social distancing requirements (15, 16). This demand for electronic health resources has provoked a revolution in the mode of health communication (16) and required relevant abilities known as eHealth literacy (EHL). EHL refers to “the ability to seek, find, understand, and appraise health information from electronic resource and apply that knowledge to solving a health problem or making a health-related decision” (17). Recently, researchers have shown that EHL has played an important role during the COVID-19 pandemic (18), and individuals with higher EHL were found to have healthier lifestyles (19). These findings have raised conjecture about this question: Can EHL benefit individuals' PA levels and engagement? If so, what is the mechanism?

Adequate EHL has long been seen as a crucial predictor of positive health outcomes (20, 21), but evidence for an association between EHL and PA seems insufficient. Some studies have confirmed a strong, positive association between EHL and PA (22–24). However, Vâjâean and Baban (25) found an inconsistent result, showing that EHL had no impact on the relationship between eHealth usage frequency and health behaviors engagement, including PA. Yet, most existing studies have neither considered PA behavior independently from other health behaviors nor measured PA intensity. Knowledge is limited regarding the relationship between EHL and PA specifically.

EHL was raised on the grounds of Health Literacy (HL) (17). Unpacking the HL concept and the HL-PA relationship provides useful background to elucidate the EHL-PA link. HL was defined as “the personal, cognitive and social skills which determine the ability of individuals to gain access to, understand, and use information to promote and maintain good health” (26). Many HL studies have been closely related to lifestyle behaviors such as PA (27). Available evidence supports the effectiveness of enhancing HL on PA (28–36), yet this evidence has been deemed inconsistent and insufficient (3, 29, 32, 37). Inconsistent findings may be because a great deal of HL research has been clinically and medically oriented (38), focusing on disease control or health indicator tracking rather than PA promotion specifically. This has created a gap in HL studies in non-medical settings (39) and regarding the general public's health efforts (1, 4, 32), where PA is a key endeavor. Additionally, literature concerning the association between HL and PA independent of other health behaviors has been extremely limited (26, 38, 40). Most studies have tested PA frequency and duration but not intensity specifically (36, 40, 41).

Literature on the relationship between HL and PA provides useful background for exploring the association between EHL and PA. However, some key issues remain unclear—how much do the HL-PA and EHL-PA relationships overlap? Which part of the HL literature could be referenced for the EHL-PA relationship? To answer these, clarifying the difference between HL and EHL has become necessary. So far, differences between EHL and HL have mostly been discussed conceptually, regarding EHL as either a type of HL in an electronic context (22, 42), or a related but distinct concept from HL (17, 43). Little empirical evidence shows how closely the two concepts are related or how distinct. Only three studies have examined the association between EHL and HL (44–46) but with inconsistent findings. This may result from advances in information technology, requiring individuals to not only handle read-only websites but also social media and mobile internet (47). EHL now includes abilities to interact with information from machine learning and artificial intelligence (48), an aspect never included in HL before. Therefore, HL and EHL should be tested separately as distinct variables, and their association is essential to be explored further.

There are two common measurement strategies for both EHL and HL, objective (skill-performance-based) and subjective (self-report-based) approaches (49). Self-reported and skill-performance measures of HL/EHL have been assumed to significantly correlate. However, several scholars (50–52) found the opposite, suggesting the two HL measurement approaches test different constructs. Unlike observable variables such as PA, HL refers to a mixture of knowledge and abilities. Ability levels can be represented by successfully completing knowledge application exams (testing application) or estimated from previous experience (reporting interpretation). There is a gap between knowing and applying, although they may positively relate. Given this, the current study separates HL into two aspects: perceived and performance. Meanwhile, a gap has also been found between perceived EHL and actual performance (53). According to Bodie and Dutta (43), actual EHL performance comprises health-related abilities and internet-related skills. The health-related abilities link to HL performance and could directly relate to health outcomes. Therefore, this study assumes (1) EHL can be jointly represented by Perceived EHL (PEHL) and HL performance (HLP), (2) HL can be jointly represented by Perceived HL (PHL) and HLP, and (3) PEHL, PHL, and HLP should be three distinct yet closely related variables.

The association between EHL/HL and PA was introduced previously. Likewise, the process through which EHL/HL affects PA should be explored. Potential mediators between HL and PA include self-efficacy (SE), social support (SS), and intention (INT) toward PA (37, 54–56). SE and SS are important components of Social Cognitive Theory (SCT) (57, 58) and are consistently related to PA (59). Meanwhile, SCT is one of the important theoretical foundations contributing to the development of EHL (17), making SCT an adoptable theory in the current study. SCT explains behavior through triadic reciprocal determinism between person, environment, and behavior factors which interact and influence each other (57, 58). Specifically, here, HL/EHL, SE, SS, and PA associate but their roles are unclear. Fortunately, the Theory of Planned Behavior (TPB) offers a framework being one of the most widely applied models for explaining informational/motivational influences on behavior (59, 60). TPB suggests an individual's behavioral INT is the proximal determinant of engaging in a specific behavior. INT is determined by one's attitude, subjective norms, and perceived behavioral control (61). INT has also been found to mediate the HL-PA relationship (36, 62, 63), making TPB another adoptable theory. However, adjustments enable better TPB application here. Regarding attitude, only one study suggested its mediating effect on HL-PA was insignificant (4). Inferring from experience that obtained health-related knowledge/abilities can hardly influence PA attitude, for example, learning more about the health benefits or technique of exercise may not make someone enjoy PA or change their feelings about it. The knowledge itself does not directly foster a passion for application. Thus, the current research decided not to consider attitude as a mediator. Subjective norms are replaced by social support per Rhodes et al. (64) and Courneya et al. (65)'s suggestion. They indicated that PA was affected by assistance from others (i.e., SS) and not capable of completely being done at will, thus when applying the TPB to exercise, SS may be superior to subjective norms for understanding exercise INTs. For perceived behavior control (PBC), Ajzen (66) identified that it contained two distinct factors: SE and controllability. Multiple studies suggest that SE tends to have a stronger impact on INT among young healthy adults (67). This is because healthy populations are less likely to face physical or environmental difficulties and more inclined to perceive full capability to achieve physical activities. Since the current study tested the proposed model among college students, it was believed that SE plays a more significant role in the current model testing.

In summary, the current research aimed to (1) explore the relationships among EHL, HL, and PA and (2) identify mediating mechanisms underlying EHL/HL-PA links. An integrated social-cognitive model was proposed and confirmed, elucidating the relationships among EHL, HL, and PA by incorporating constructs from SCT and TPB (including SE, SS, and INT). This integrated model examines factors potentially explaining and mediating EHL/HL-PA links. It is well-suited for growing research focused on how EHL/HL influences health behaviors, while a large portion of them examined the role of SE, SS, and behavioral INT. Predictions were tested using a three-wave prospective survey of Chinese college students. This population was targeted for several reasons: (1) As major internet users who explore emerging technologies, investigating EHL impacts in this adept, educated population is essential amidst digital health evolution; (2) Data were collected during COVID-19 when students were motivated to rebuild physical activity routines after restrictions, given their confidence in fitness and autonomy in health decisions; (3) Longitudinal access enabled feasible multi-wave surveying. This study can help scholars and practitioners of health promotion to better understand EHL and tailor eHealth interventions for Chinese college students. In the long term, the findings could provide valuable references for policymakers developing strategies to promote both PA and EHL in China.

### Hypothesis

**Figure 1:**
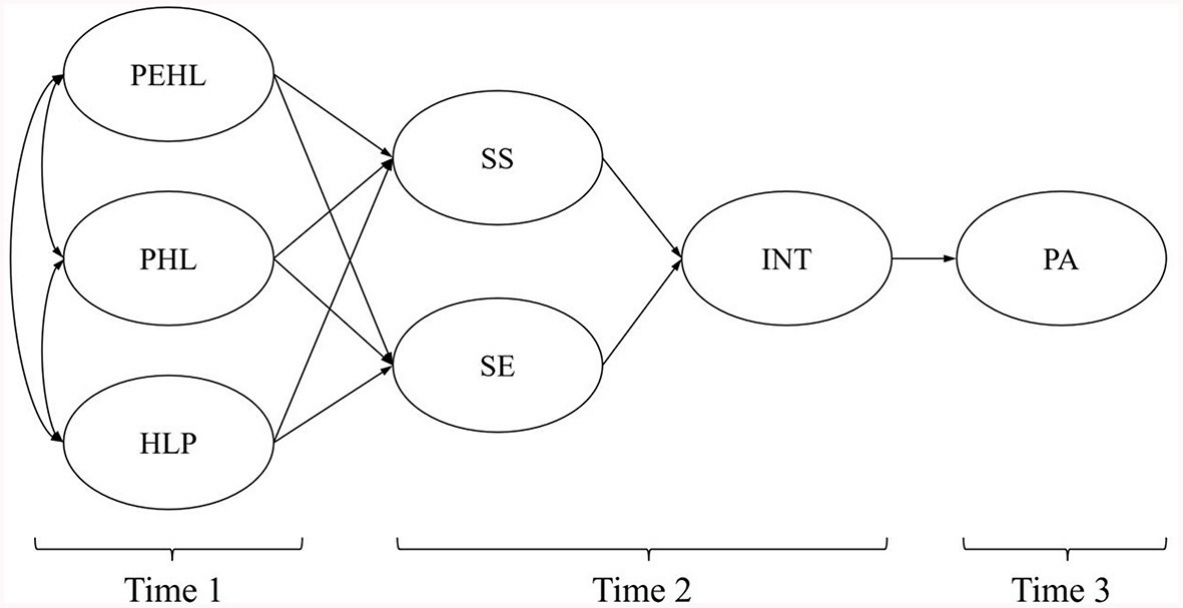
The hypothesized model. PEHL, Perceived eHealth Literacy; PHL, Perceived Health Literacy; HLP, Health Literacy Performance; PA, Physical Activity; SE, Self-efficacy for PA; SS, Social support for PA; INT, Intention for PA.

In total, 12 direct paths were hypothesized in the proposed model (see Figure 1). It was expected that

*Hypothesis 1*

a. PEHL, PHL, and HLP (Time 1) would positively correlate with each other;
b. PEHL, PHL, and HLP (Time 1) would predict SS (Time 2);
c. PEHL, PHL, and HLP (Time 1) would predict SE (Time 2);
d. SS and SE (Time 2) would predict INT (Time 2); and
e. INT (Time 2) would predict PA (Time 3).

In total, 12 indirect mediating relationships were hypothesized in the proposed model. It was expected that

*Hypothesis 2*

a. SS would mediate the effect of PEHL, PHL, and HLP (Time 1) on INT (Time 2) respectively; and
b. SE would mediate the effect of PEHL, PHL, and HLP (Time 1) on INT (Time 2) respectively.

It was also expected that

*Hypothesis 3*

a. SS and INT (Time 2) would jointly mediate the effect of PEHL, PHL, and HLP (Time 1) on PA (Time 3) respectively; and
b. SE and INT (Time 2) would jointly mediate the effect of PEHL, PHL, and HLP (Time 1) on PA (Time 3) respectively.

## Method

### Participants

This study used a three-wave prospective longitudinal design. Ethical approval was obtained from the Research Ethics Committee of Hong Kong Baptist University. Convenience sampling (68) was applied to recruit participants from four representative Chinese cities, Beijing, Xiamen, Kunming, and Yinchuan. These four cities were selected based on the consideration of the geographic location (i.e., north, southeast, southwest, and northwest), political status (i.e., country capital, provincial capital, and prefecture-level city), and economic status (i.e., high, medium, and low) of Chinese cities (69, 70). Furthermore, the choice of those four cities was also made with the issues of “convenience and feasibility” (71). Considering the item-to-response ratios of at least 1: 10 (72) and the recommendation for a minimum sample size of 100 to 150 in structural equation modeling (73), at least 240 observations were required (1:10 item-to-response ratio) in this study. Ultimately, 947 college students completed the three-wave survey (following the “rule of thumb” and depending on the natural history of the condition under study) (74), offering an adequate sample size. Inclusion criteria were: (1) experience using eHealth websites/tools; (2) sufficient Chinese language skills; and (3) informed consent to participate. In total, 1,342 participants completed the baseline survey and 294 and 101 participants dropped out at the second and third waves, respectively. Finally, 947 participants completed all three waves and were included in the data analysis. All the measures were administered in Chinese.

### Measures

#### Demographic information

Participants' age, gender, major, region, and year of college study were collected at the baseline survey.

#### Perceived eHealth literacy

The eHealth literacy scale in Web 3.0 context (eHLS-Web3.0) developed by Liu et al. (48) consists of 24 items sorted into three dimensions: acquisition (8 items), verification (6 items), and application (10 items). An example dialogue question is “I know how to make use of the records on the eHealth tools to provide reference for my daily health management.” Responses are indicated on a 5-point Likert scale from 1 (strongly disagree) to 5 (strongly agree). The eHLS-Web3.0 was developed on the basis of a literature review and interviews, with validity confirmed through exploratory and confirmatory factor analysis. Validity, reliability, and measurement invariance have been established among college students. Compared to previous EHL tools, the eHLS-Web3.0 assesses modern eHealth usage behaviors including social media and mobile technology. As a recently developed measurement, the eHLS-wEB3.0 demonstrated high internal consistency (Cronbach's α) of 0.971 for the full scale and 0.913–0.962 for sub-scales in a Chinese college sample (48). In the present study, the eHLS-Web3.0 also showed good internal consistency (Cronbach's α = 0.977).

#### Perceived health literacy

The three-item Health Literacy Screening Questionnaire (75, 76) was chosen to assess PHL. It comprises 3 items in a 5-point Likert format: (1) How often do you need someone to read hospital materials? (2) How often do you have problems learning about your medical condition because of difficulty reading hospital materials? (3) How confident are you filling out medical forms by yourself? Responses are indicated on a 5-point Likert scale from 1 (strongly disagree) to 5 (strongly agree). Per developer recommendations, the first two items were reverse coded and the third retained the original, so higher scores indicated greater PHL (75, 76). This widely used tool has demonstrated validity and reliability across diverse groups and against other common HL measures (76–79). Two health promotion PhD students initially translated the original questionnaire into Chinese. Back-translation was done by a senior English teacher fluent in Chinese. In the current study, the Chinese version showed good internal consistency among college students (Cronbach's α = 0.935).

#### Health literacy performance

The short-form Mandarin Health Literacy Scale (s-MHLS) (80) was chosen to test participants' HLP (application of HL knowledge). It is an 11-item performance-based test for measuring Taiwanese adults' HL, including Cloze-type questions simulating patient-physician dialogue (4 items), and a prescription with comprehension questions for assessing abilities to understand textual and numeric information (6 items). An example dialogue question is “Doctor, the big toe on my right foot has been in pain and [blank] for 4 or 5 days, and it's getting worse.” with options: (a) Fat; (b) Swelling; and (c) Dehydration. An example prescription question is “How much of this medicine should the patient take each time?” with options: (a) 1/2 Tablet; (b) 1 Tablet; and (c) 5 Tablet, along with relative information presented on the prescription “Usage & Dosage: Take orally. 1/2 tablet per day, 30 min before breakfast.” Correct responses receive 1 point and incorrect 0 points. Scores are summed, with higher scores indicating better HLP. The s-MHLS was adapted from the Mandarin Health Literacy Scale (81) and developed based on Nutbeam's health literacy framework (26). Both the original and short-form versions demonstrated good validity and reliability across diverse population groups (80–83). For Taiwanese adults, the s-MHLS has a 0.94 internal consistency. In the present study, acceptable internal consistency was found (Cronbach's α = 0.622).

#### Self-efficacy for physical activity

A behavior-specific scale drawn from Liang et al. (84)'s paper was selected. That scale measured SE specifically for PA among Chinese college students. It was translated from Luszczynska and Sutton (85)'s research and tests SE for PA with the stem “I am certain that…” followed by 5 items for PA such as “…I can be physically active permanently at a minimum of 5 days a week for 30 min.” The answer was indicated on a 5-point Likert scale, ranging from do not agree at all, “1,” to agree completely, “5,” This scale showed good validity and reliability in Chinese college students (Cronbach's α = 0.88) (84, 85). In the present study, good internal consistency was found (Cronbach's α = 0.964).

#### Social support for physical activity

A behavior-specific scale drawn from Liang et al. (84)'s paper was used. That scale measured social support specifically for PA among Chinese college students. It was translated from Jackson et al. (86)'s research and tests social support for PA with the stem as “How do you perceive your environment…” followed by 3 items for PA such as “…People like my classmates and friends help me to stay physically active.” The answer was indicated on a 5-point Likert scale, ranging from do not agree at all, “1,” to agree completely, “5.” This scale showed good validity and reliability in Chinese college students (Cronbach's α = 0.72) (84, 86). In the present study, good internal consistency was found (Cronbach's α = 0.899).

#### Intention for physical activity

A behavior-specific scale drawn from Liang et al. (84)'s paper was used. That scale measured intention specifically for PA among Chinese college students. It was translated from Lippke et al. (87)'s article, and includes the stem of “On 5 days a week for 30 min (or a minimum of 2.5 h per week), I have the intention to perform…” followed by 3 items for different intensity levels of PA such as “strenuous PA,” “moderate PA,” and “mild PA.” The answer was indicated on a 5-point Likert scale, ranging from do not agree at all, “1,” to agree completely, “5.” The validity and reliability were tested in Chinese college students (Cronbach's α = 0.34) (84, 87). In the present study, good internal consistency was found (Cronbach's α = 0.958).

#### Physical activity

PA was measured by using the Chinese short version of the International Physical Activity Questionnaire (IPAQ-C) [International Physical Activity Questionnaire, (88); IPAQ-C, (89)]. IPAQ-C consists of 6 items, which ask participants to report their PA level with three intensities (vigorous, moderate, and mild). Corresponding to each intensity, participants are asked to indicate how often per week and how long each time for performing these activities in the past seven days. This questionnaire includes items such as “During the last 7 days, on how many days did you engage in moderate physical activities like carrying light loads, bicycling at a regular pace, or doubles tennis? (Do not include easy walking),” and “How much time did you usually spend doing moderate physical activities on one of those days?” Based on these indications, the aggregate amount of time for total PA and intensity of PA (in minutes per week) were calculated for the past seven days (89). As per the IPAQ protocol, we calculated the IPAQ result as a continuous variable. Participants who satisfied the criteria of either (a) high-intensity PA on at least 3 days achieving a minimum total PA of at least 1,500 MET-minutes/week (one MET is what a participant expended when he/she was at rest); or (b) 7 or more days of any category of PA achieving a minimum total PA of at least 3,000 MET-minutes/week were labeled as “high-intensity PA group.” Participants who satisfied the criteria of either (a) 3 or more days of high-intensity PA of at least 20 min per day; (b) 5 or more days of mid-intensity PA and/or low-intensity PA of at least 30 min per day; or (c) 5 or more days of any combination of any category of PA achieving a minimum total PA of at least 600 MET-minutes/week were labeled as “mid-intensity PA group.” The rest participants who did not meet any criteria listed before were labeled as “low-intensity PA group” (90).

### Procedures

Informed consent was obtained prior to the survey. Questionnaire items were distributed to participants online. Demographics, PEHL, PHL, and HLP were collected at baseline. SS, SE, and INT were collected at the 2-month follow-up. PA was collected at the 4-month follow-up occasions. Specifically, data collection occurred in three waves: September 2020 (*n* = 1,342), November 2020 (*n* = 1,048), and January 2021 (*n* = 947). According to the Lancet, China had brought COVID-19 to a very low level and managed to control the pandemic effectively by October 2020 (91). The coronavirus tracking report also showed stable, low transmission in China during data collection, with a daily average of 25 reported cases on 31 August 2020 and 28 cases on 9 February 2021 (92). The “Zero-COVID strategy” was applied at that time, involving large-scale nucleic acid testing and domestic travel restrictions from high-risk areas, but did not forbid outdoor activities (93). Although social distancing may have potentially influenced daily PA, participants were likely resuming normal living and engaging in some PA during the study period. A flow chart of participant recruitment is provided in the Appendix.

### Statistical analysis

A conceptual model was constructed based on theory and evidence. The distribution of the data was examined to determine the level of skewness and kurtosis, in combination with the means and standard deviations. Skewed data were log-transformed and replaced with median values (interquartile range). Means and standard deviations were calculated for the variables. Chi-square tests were performed to compare baseline characteristics across the PA groups. One-way ANOVA was applied to check whether the EHL level was invariant across gender, region, major, and year of study. Pearson correlation coefficients were also calculated among the variables.

Path analysis was used to confirm the proposed model quantitatively by the maximum likelihood estimation approach via Mplus 7 (94). General accepted model fit indexes were adopted. The chi-square statistic (χ^2^) was used to test the model's overall goodness of fit (95). Multiple model fit indices then were examined further, including the comparative fit index (CFI) (96) and the Tucker-Lewis index (TLI) (97), with a cutoff value of around 0.90 and above recommended to indicate a satisfactory fit for the CFI and TLI (98); the standardized root mean residual (SRMR) (96), whose values near 0.08 indicate adequate model fit; and the root mean square error of approximation (RMSEA) and its 90% confidence interval (99), for which values <0.08 was indicative of good fit. The strength of relationships among variables was calculated using standardized path coefficients.

Furthermore, in order to examine the indirect effects of distal variables of the proposed model in explaining PA behavior, the bootstrapping method was used with 5,000 bootstrap samples (100). The confirmation of an indirect effect of a predictor variable (e.g., SE) on an outcome variable (e.g., PA behavior) is by a confidence interval of the estimate which does not contain zero (100).

## Results

### Sample characteristics and correlations of variables

A total of 947 participants (500 women, 447 men; 19.87 ± 1.68 yrs.) were included in the data analysis. A summary of participants' demographic information is listed in Table 1, showing data on PA and socio-demographic factors. The Chi-square tests indicated that the PA levels of students from various regions were slightly different (χ^2^ = 19.826, *P* = 0.03) but no significant differences were found for gender, major, and year of study. The EHL level of participants was found to be invariant across gender (*P* = 0.88), major (*P* = 0.05), region (*P* = 0.44), and year of study (*P* = 0.99).

**Table 1:** Sample characteristics and variation in PA (*n* = 947).

**Demographic information**	**Total**		**High-intensity group**		**Mid-intensity group**		**Low-intensity group**		**χ^2^/df**
	***n***	**Frequency (%)** ^a^	***n***	**Frequency (%)**	***n***	**Frequency (%)**	***n***	**Frequency (%)**	
**Gender**									0.935(2) (*P =* 0.627)
Male	447	(47.2)	113	(25.3)	171	(38.3)	163	(36.5)	
Female	500	(52.8)	114	(22.8)	203	(40.6)	183	(36.6)	
**Major**									4.677(4) (*P =* 0.322)
Medical	54	(5.7)	10	(18.5)	28	(51.9)	16	(29.6)	
Sport	142	(15.0)	39	(27.5)	54	(38.0)	49	(34.5)	
Non-health related	751	(79.3)	178	(23.7)	292	(38.9)	281	(37.4)	
**Region**									19.826(6) (*P =* 0.003)
Beijing	144	(15.2)	39	(27.1)	66	(45.8)	39	(27.1)	
Xiamen	357	(37.7)	76	(21.3)	130	(36.4)	151	(42.3)	
Kunming	281	(29.7)	75	(26.7)	122	(43.4)	84	(29.9)	
Ningxia	165	(17.4)	37	(22.4)	56	(33.9)	72	(43.6)	
**Year of study**									9.087(6) (*P =* 0.169)
Year 1	41	(4.3)	13	(31.7)	20	(48.8)	8	(19.5)	
Year 2	686	(72.4)	157	(22.9)	262	(38.2)	267	(38.9)	
Year 3	122	(12.9)	33	(27.0)	52	(42.6)	37	(30.3)	
Year 4	98	(10.3)	24	(24.5)	40	(40.8)	34	(34.7)	

^a^column % for each gender, major, region.

The correlation matrix of the study variables is shown in Table 2. PEHL was found to be positively related to PHL, and PA indicators including SE, SS, and INT. PA was also positively associated with HLP, SE, and INT. In addition, there were significant positive associations among the three PA indicators, SE, SS, and INT.

**Table 2:** Bivariate correlations for the study variables (*n* = 947).

	**PEHL (T1)**	**PHL (T1)**	**HLP (T1)**	**PA (T3)**	**SE (T2)**	**SS (T2)**	**INT (T2)**
**PEHL** **(T1)**	1						
**PHL** **(T1)**	0.427 *P <* 0.001	1					
**HLP** **(T1)**	0.099 *P =* 0.002	−0.147 *P <* 0.001	1				
**PA** **(T3)**	0.091 *P =* 0.005	−0.082 *P =* 0.012	0.144 *P <* 0.001	1			
**SE** **(T2)**	0.569 *P <* 0.001	0.297 *P <* 0.001	0.017 *P =* 0.598	0.182 *P <* 0.001	1		
**SS** **(T2)**	0.526 *P <* 0.001	0.372 *P <* 0.001	−0.074 *P =* 0.022	0.110 *P =* 0.001	0.651 *P <* 0.001	1	
**INT** **(T2)**	0.510 *P <* 0.001	0.305 *P <* 0.001	0.026 *P =* 0.423	0.168 *P <* 0.001	0.834 *P <* 0.001	0.669 *P <* 0.001	1
**Mean**	84.12	9.23	9.50	2,016.65	17.54	20.04	10.65
**SD**	17.64	2.90	1.65	2,470.28	4.12	5.07	2.52
**α**	0.977	0.935	-	-	0.964	0.899	0.958

PEHL, perceived eHealth literacy; PHL, perceived health literacy; HLP, health literacy actual performance; PA, physical activity; SE, self-efficacy for PA; SS, social support for PA; INT, intention for PA; T1, Time 1; T2, Time 2; T3, Time 3; SD, standard deviation; α, Cronbach's alpha for measurement's internal consistency reliability.

### Model fit and path analysis

**Figure 2:**
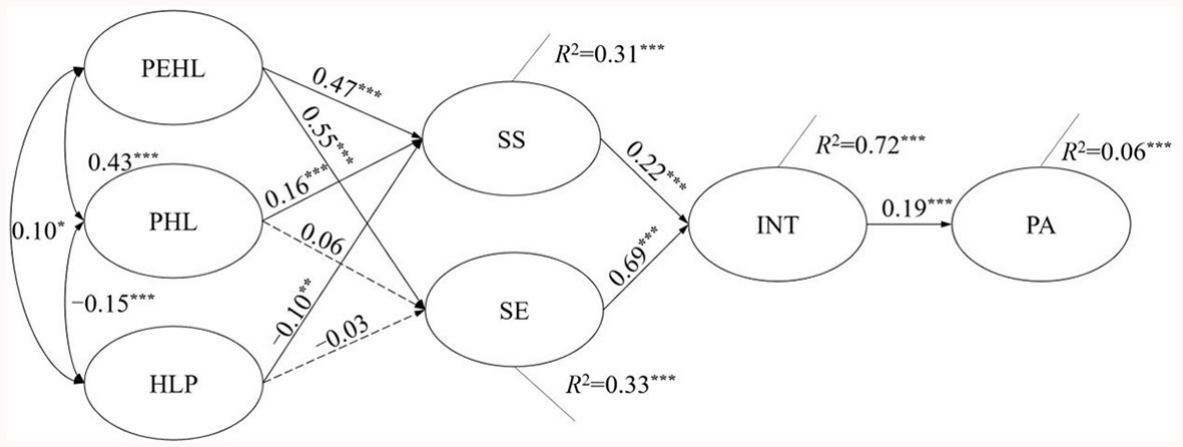
Standardized parameter estimates for the proposed model. ^*^p < 0.05; ^**^p < 0.01; ^**^p < 0.001.

The result of the path analysis is shown in Figure 2. An adequate good-to-fit model was indicated with the fit indices as χ^2^ = 6.808, χ^2^/df = 3.404 (*P* = 0.033), CFI = 0.998, TLI = 0.981, RMSEA = 0.050, SRMR = 0.008.

Considering the association among PEHL, PHL, and HLP, it was found that PEHL remained a direct and significant association with PHL (β = 0.43, *P* < 0.001) and a mild positive association with HLP (β = 0.10, *P* = 0.02). A negative relationship was found between PHL and HLP (β = −0.15, *P* < 0.001).

As per the tests of hypothesized effects of the model demonstrated in Figure 2, PEHL had a significantly positive effect on SE (β = 0.55, *P* < 0.001) and SS (β = 0.47, *P* < 0.001). A significantly positive effect of PHL was found on SS (β = 0.16, *P* < 0.01), and the effect of PHL on SE was not supported. A mild negative association between HLP and SS (β = −0.10, *P* = 0.001) was indicated, and the correlation between HLP and SE demonstrated an insignificant result. As expected, both SS (β = 0.22, *P* < 0.01) and SE (β = 0.69, *P* < 0.001) had a significant positive effect on INT, while the effect of SE was stronger than the SS. Of interest, INT was then found to significantly connect to PA (β = 0.19, *P* < 0.001).

For the percentage of variance explained in the model, the relationships proposed in the model explained 31.2% of social support (*R*^2^ = 0.31), 32.8% of self-efficacy (*R*^2^ = 0.33), 72.4% of intention (*R*^2^ = 0.72), and 6.3% of PA (*R*^2^ = 0.06).

### Total effects, indirect effects, and direct effect

The indirect effects of the model are exhibited in Table 3. As for the path of PEHL to INT, the total effect of PEHL on INT (β = 0.465, *P* < 0.001) was confirmed. SS (β = 0.103, *P* < 0.001) and SE (β = 0.377, *P* < 0.001) were found to significantly mediate the relationship between PEHL and INT. The direct effect of PEHL on INT was not significant (β = −0.014, *P* = 0.591), which demonstrated the full mediating effects of SS and SE on the path from PEHL to INT. As for the path of PHL to INT, the total effect of PHL on INT (β = 0.105, *P* < 0.05) was confirmed. SS (β = 0.034, *P* < 0.05) was found to significantly mediate the relationship between PHL and INT, while the mediating effect of SE (β = 0.041, *P* = 0.096) on the same relationship was not supported. The direct effect of PHL on INT was not significant (β = 0.029, *P* = 0.192), which means SS played a completely mediating role on the path of PHL-INT. As for the path of HLP to INT, the total effect of HLP on INT (β = −0.005, *P* = 0.892) was not significant, thus neither SE nor SS can mediate the relationship between HLP and INT.

**Table 3:** Total effects, indirect effects, and direct effects of the hypothesized model (*n* = 947).

	**PA INT**	**PA**
	**Est (95% CI)**	**Est (95% CI)**
**Total effects**		
PEHL → INT	0.465 (0.352, 0.568) *P <* 0.001	
PHL → INT	0.105 (0.010, 0.202) *P =* 0.006	
HLP → INT	−0.005 (−0.095, 0.080) *P =* 0.892	
PEHL → PA		0.132 (0.046, 0.222) *P <* 0.001
PHL → PA		−0.121 (−0.219, −0.024) *P =* 0.002
HLP → PA		0.113 (0.040, 0.185) *P <* 0.001
**Indirect effects**		
Total PEHL → INT	0.479 (0.390, 0.565) *P <* 0.001	
PEHL → SS → INT	0.103 (0.058, 0.159) *P <* 0.001	
PEHL → SE → INT	0.377 (0.292, 0.461) *P <* 0.001	
Total PHL → INT	0.076 (−0.003, 0.156) *P =* 0.014	
PHL → SS → INT	0.034 (0.012, 0.074) *P =* 0.002	
PHL → SE → INT	0.041 (−0.021, 0.106) *P =* 0.096	
Total HLP → INT	−0.041 (−0.110, 0.023) *P =* 0.106	
HLP → SS → INT	−0.021 (−0.045, −0.006) *P =* 0.003	
HLP → SE → INT	−0.019 (−0.074, 0.032) *P =* 0.339	
Total PEHL → PA		0.086 (0.045, 0.138) *P <* 0.001
PEHL → SS → INT → PA		0.019 (0.009, 0.034) *P <* 0.001
PEHL → SE → INT → PA		0.070 (0.037, 0.111) *P <* 0.001
Total PHL → PA		0.019 (0.002, 0.043) *P =* 0.014
PHL → SS → INT → PA		0.006 (0.002, 0.015) *P =* 0.005
PHL → SE → INT → PA		0.008 (−0.004, 0.022) *P =* 0.117
Total HLP → PA		−0.001 (−0.018, 0.016) *P =* 0.894
HLP → SS → INT → PA		−0.004 (−0.009, −0.001) *P =* 0.006
HLP → SE → INT → PA		−0.004 (−0.015, 0.006) *P =* 0.351
PEHL → INT	−0.014 (−0.081, 0.057) *P =* 0.591	
PHL → INT	0.029 (−0.026, 0.088) *P =* 0.192	
HLP → INT	0.036 (−0.012, 0.084) *P =* 0.058	
PEHL → PA		0.046 (−0.051, 0.142) *P =* 0.224
PHL → PA		−0.141 (−0.243, −0.045) *P <* 0.001
HLP → PA		0.114 (0.043, 0.185) *P <* 0.001

PEHL, perceived eHealth literacy; PHL, perceived health literacy; HLP, health literacy actual performance; PA, physical activity; SE, self-efficacy for PA; SS, social support for PA; INT, intention for PA.

As for the path of PEHL to PA, the total effect of PEHL on PA (β = 0.132, *P* < 0.001) was significant. SS and INT (β = 0.019, *P* < 0.001) jointly mediated the effect of PEHL on PA as did SE and INT (β = 0.070, *P* < 0.001). The direct effect of PEHL on PA was not significant (β = 0.046, *P* = 0.224), which means SS-INT and SE-INT played completely mediating roles on the path of PEHL-PA. As for the path of PHL to PA, the total effect of PHL on PA (β = −0.121, *P* < 0.05) was significant. SS and INT (β = 0.006, *P* < 0.05) jointly mediated the effect of PHL on PA, while the mediating effect of SE together with INT was not significant (β = 0.008, *P* = 0.117). The direct effect of PHL on PA was significant (β = −0.141, *P* < 0.001), which means SS and INT jointly played a partial mediating role on the path of PHL-PA. As for the path of HLP to PA, the total effect of HLP on PA (β = 0.113, *P* < 0.001) was significant. SS and INT (β = −0.004, *P* < 0.05) jointly mediated the effect of HLP on PA, while the mediating effect of SE together with INT was not significant (β = −0.004, *P* = 0.351). The direct effect of HLP on PA was significant (β = 0.114, *P* < 0.001), which means SS and INT jointly played a partial mediating role on the path of HLP-PA.

## Discussion

The current research examined the relationship among EHL, HL, and PA, and identified the mediating role of SS, SE, and INT in the aforementioned association. The study was conducted when the COVID-19 pandemic was under control (91–93). By reviewing existing measures, EHL and HL were deconstructed into PEHL, PHL, and HLP. Relationships among PEHL, PHL, and HLP were confirmed, with a negative PHL-HLP association. A model was proposed and tested based on the hypothesis that PEHL/PHL/HLP would indirectly and positively associate with PA: PEHL positively correlated with SE and SS; PHL positively correlated with SS; HLP negatively correlated with SS on a mild level; SS and SE positively associated with INT; and INT significantly predicted PA. For mediation, SS significantly mediated PEHL/PH-INT; SE only mediated PEHL-INT; SS and INT jointly mediated PEHL/PHL/HLP-PA; and SE and INT jointly mediated PEHL-PA. The findings are discussed in light of the data analysis.

### Relationships among perceived eHealth literacy, perceived health literacy, and health literacy performance

The current study found those with higher PEHL tend to have better PHL, though moderately. This confirms EHL and HL should be tested as distinct variables, providing evidence for past qualitative work suggesting EHL and HL represent different abilities in different contexts rather than similar skills (47). Meanwhile, the association between PEHL and HLP was weak. This aligns with Richtering et al. (44), who deconstructed EHL into four aspects: usefulness, critical evaluation, navigating resources, and skill to use. The former two represent perceived EHL; the latter two, actual performance. No significant association occurred between the perceived and performance aspects, suggesting that interpreted knowledge is independent from the application of knowledge and skill in the health domain. Also, most HL instruments were developed for clinical context, whereas EHL represents a public health focus. The EHL measurement used in this study also evaluates mobile internet usage, while most HL instruments did not account for technological context. These differences in intended context and measurement approaches may explain the non-significant association found between PEHL and HLP.

Inconsistent with the hypotheses, higher PHL was associated with lower HLP scores. However, past research has also found weak PHL-HLP links (50–52). This may result from the college student sample, who tend to overestimate their HL due to high education (101, 102), despite typically limited clinical knowledge without specific training or experiences (103). With experience, estimations would likely be lower but more accurate. Additionally, as suggested by Cress et al. (104), external determinants such as depressive symptoms could negatively affect self-perceived abilities, while the performance-based task can reflect actual ability level more objectively. Since this study was conducted during the COVID-19 pandemic, students with clinical experience may be more susceptible to depression (105) and therefore have a more negative perception of their HL competency, while still performing adequately on HL assessments. Further studies incorporating mental health indicators are warranted to more comprehensively examine this relationship.

### Path analysis of the hypothesized model

This study applied an integrated model based on SCT and TPB to explore the relationships among EHL, HL, and PA. Similar investigations using TPB and SCT have occurred in previous HL studies (106–109), mostly in Western contexts without applying assumptions to explain PA. Thus, this exploration provides further explanation and a deeper understanding of the effect of EHL/HL on PA from the perspective of TPB and SCT, and from a diverse cultural background.

The proposed PEHL-PA path was confirmed, aligning with a study of EHL and human papilloma virus (HPV) vaccination (health behavior) that found EHL significantly predicted health behavioral INT and subsequent health behavior (110). Similarly, INT-PA effects were weak here, supporting the INT-PA gap (111) and suggesting INT changes could be a primary EHL intervention target since EHL involves applying eHealth information. EHL-based INT-translation follow-up is worth considering. As expected, SS and SE positively predicted INT, aligning with previous theories (52, 64, 69) and studies (108, 109).

The proposed positive path from PEHL to SE was confirmed. One explanation may be that SE enhances openness to adopting new electronic technologies (112). In addition, SE facilitates the uptake and use of eHealth devices. However, PHL and HLP did not significantly associate with SE, counter to some scholars (4, 54, 113, 114). However, those studies involved older adults and patients vs. the young, healthy sample here. Research on healthy Latinas (37) aligns with the current study, explaining better HL allows more realistic PA SE perceptions. Additionally, research among heart failure patients (115) found that HL and heart failure knowledge were not related to SE for heart failure self-care, suggesting knowledge and behavioral efficacy are weakly associated. As college students are prone to perceiving full physical ability regardless of HL (67), the non-significant PHL/HLP-SE correlations make sense. PEHL/PHL/HLP relationships with SS were confirmed, aligning with other studies (106, 107, 116). The slight negative HLP-SS association replicated Lee et al. (116) and Lora et al. (109), where individuals with lower HL sought more help and support, especially informational and emotional. Therefore, providing SS could buffer the effects of lower HL and increase PA intention among students when promoting a PA-supportive university environment. Practical initiatives are suggested, such as offering group exercise opportunities or providing access to workout buddies or groups.

### Mediating role of social support, self-efficacy, and intention

A bootstrap method was used to provide support for the mediating effect of variables in the proposed model. SE and SS were found to have a complete mediating effect on the PEHL-INT link, likewise, SE had no mediating effect on other associations. SE-INT and SS-INT fully mediated PEHL-PA. SS-INT fully mediated PHL-INT and partially mediated PHL/HLP-PA. Hence, potential mediators in the relationships of PHL/HLP and PA should be further explored. SE-INT had no effect on other associations.

Compared to SS, SE played a more influential role in EHL-related associations. This may be because adequate EHL helps individuals handle chaotic online health data, avoid uncertainty-induced lack of control, and be willing to engage in health behaviors during the COVID-19 pandemic (16). Britt et al. (110) also suggested that behavior-related SE is a crucial factor in the decision-making process from EHL toward health behavior. Compared with SE, SS played a more active role in HL-PA links. A qualitative study (117) suggests Asians rarely integrate PA into their lifestyles, instead, PA is more often seen as an opportunity for social activities. Thus, PA-based social activities were popular among individuals with adequate HL. Hence, facilitating group-based PA through communities and networks would be an effective way to promote PA among Asian people.

Overall, the examination of indirect effects in the current study provides understanding of the relationships among e-Health Literacy (EHL), Health Literacy (HL), and PA. The findings specifically highlights that self-efficacy (SE) and social support (SS) play different roles in HL-PA and EHL-PA paths, which suggests a new underlying mechanism for constructing EHL and HL interventions in future studies.

### Practical implication

By integrating SCT and TPB, this research contributes to theorizing EHL's role in promoting PA. It provides additional evidence supporting previous SCT and TPB assumptions about health behavior change, specifically among Chinese college students. The confirmed model offers new insight into how EHL/HL can differently influence PA. Related interventions could apply these findings to provide empirical verification. The model could be expanded to explore EHL's effect on other health behaviors. As EHL did not largely predict PA as hypothesized, exploring additional intention-behavior factors could expand the model to better explain this relationship and inform future interventions targeting health promotion among Chinese students. Most significantly, identifying mediators of the EHL-PA relationship provides novel insights to inform health promotion initiatives targeting PA engagement. The potential social impact is multi-faceted, as individuals can apply the framework to improve their PA while scholars, professionals, and policymakers gain new evidence-based levers such as SE and SS for intervention and policies. This study also highlights differential SE and SS roles in HL-PA and EHL-PA links. These not only suggest novel approaches to frame interventions but may also inform strategies and policies to promote health behaviors in China. To our knowledge, this is the first empirical HL and EHL comparison in a Chinese context. The content deconstruction, mixed subjective-objective measurement approach, and new information on constructing training provide valuable insights.

### Limitations and future directions

The current study has several limitations. First, the generalizability of research findings might be hindered by the sampling method. As a result, a stratified sampling approach is desirable in future. Second, the results are only based on the responses among Chinese college students, therefore its application in other groups or areas needs to be examined. Third, online self-report questionnaires were applied, which may result in unreliability and inaccuracy because of the inherent drawbacks of self-reporting (i.e., recall bias, over- or under-reporting, non-response error, and social desirability) (118, 119). Fourth, it was valuable that the findings give new insights into the antecedents of behavior intention (>60%), yet the whole model only explained 6% of PA, hinting that more components and covariates need to be identified in the future (e.g., volitional factors between intention and behavior) (120). Fifth, a rapid screening tool for PHL (the three-item health literacy screening questionnaire) was used in the current study so that the number of items in the whole questionnaire could be restricted in a reasonable amount. However, this tool did not cover HL in the area of health promotion and PA, which may have biased the result. A more specific comparison between EHL and HL should be conducted with both EHL and HL tools covering the health promotion area. Sixth, the variables were assessed only at the specific time points indicated in Figures 1, 2, rather than at each time point. Collecting data for all variables at each measurement occasion could have enabled more sophisticated analysis, such as time-varying covariate or lagged effects modeling, which may have provided additional insights into relationships between the constructs over time. Finally, the current research proposed the model on the basis of SCT and TPB, while there may be some potential mediators for the HL, EHL, and PA relationships not inclusive in these two theories. Factors beyond internet usage may also predict EHL which had not been considered in the current research. Additionally, the current model did not consider the attitude component of TPB. Future research in this area is warranted.

## Conclusion

An integrated social-cognitive model based on SCT and TPB was proposed and tested in the current study to explore the relationships among EHL, HL, and PA. HL and EHL were destructed into two dimensions, personal interpretation and actual performance. Those two dimensions were found to be independent from each other. The current study suggested that those two dimensions should be measured simultaneously in future HL/EHL-targeted research for better representative individuals' EHL or HL levels. The model testing results provided insight into the explanation for the indirect effects of EHL/HL on PA. In the model, SE was found to be a more effective mediator than SS in the relationship from EHL to PA, and SS was found to play a more active role than SE in the relationship from HL to PA. Ideally, an interventional study using a randomized controlled trial design is needed to further investigate the applicability of the tested model in the current research.

## Scope statement

The spread of COVID-19 has led to a decrease in physical activity (PA) while raising the demand for electronic resources. eHealth literacy (EHL) is expected to play an important role in responding to online health information and taking appropriate activities to stay health. Yet few studies have explored the mechanism for EHL impacting PA has been conducted. In addition, although EHL was raised on the grounds of health literacy (HL), few empirical studies were found to examine the association between them. This study aimed to explore the relationships among HL, EHL, and PA. An integrated social-cognitive model was proposed and tested, and multiple mediating mechanisms under the relationships among EHL, HL, and PA were identified. The findings specifically highlighted that self-efficacy and social support played different roles in the EHL/HL-PA relationships, and suggested a new underlying mechanism for constructing further EHL/HL interventions. EHL is a crucial ability closely related to digital public health. Previous research proved that adequate EHL can not only have a positive effect on MH by protecting people from unreliable or poor-quality health information but is also significantly linked to healthy behavior engagement. In that case, we believe that this paper will be of interest to the readership of your journal.

## Data availability statement

The original contributions presented in the study are included in the article/supplementary material, further inquiries can be directed to the corresponding author.

## Ethics statement

The studies involving humans were approved by Research Ethics Committee of Hong Kong Baptist University. The studies were conducted in accordance with the local legislation and institutional requirements. The participants provided their written informed consent to participate in this study.

## Author contributions

HL: Data curation, Formal analysis, Investigation, Methodology, Project administration, Validation, Writing—original draft, Writing—review & editing. BC: Methodology, Project administration, Supervision, Writing—review & editing. HH: Project administration, Supervision, Writing—review & editing. YH: Methodology, Project administration, Supervision, Writing—review & editing. WL: Data curation, Investigation, Methodology, Validation, Writing—review & editing. RW: Project administration, Supervision, Validation, Writing—review & editing.
