# Supplementary figures and images for: Prospective association of eHealth literacy and health literacy with physical activity among Chinese college students: a multiple mediation analysis
Source: Front Public Health. 2024 Feb 8;12:1275691. doi: 10.3389/fpubh.2024.1275691 (PMC10881736; doi:10.3389/fpubh.2024.1275691)

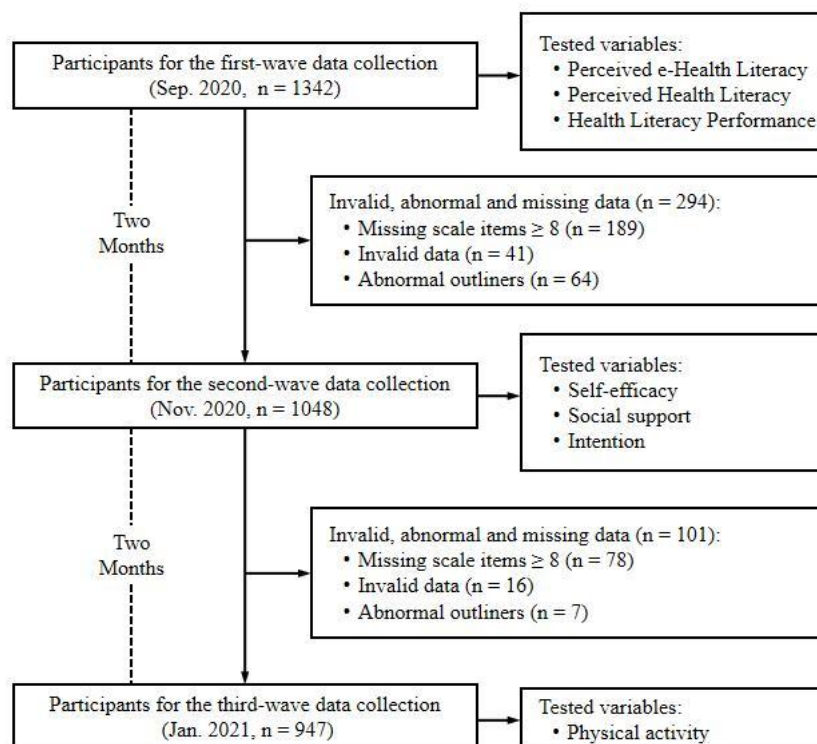

**Appendix.** Flow chart of participant recruitment

Supplement: Supplementary file 1 [file Image_1.PDF]
